# Oral health knowledge, literacy and behavior of pregnant women: a qualitative study in a northeastern province of Thailand

**DOI:** 10.1186/s12903-024-04414-3

**Published:** 2024-06-04

**Authors:** Nitikorn Phoosuwan, Pimchanok Bunnatee, Pranee C. Lundberg

**Affiliations:** 1 Department of Public Health and Caring Sciences, Faculty of Medicine, Uppsala University, Box 564, Uppsala, SE-751 22 Sweden; 2 Department of Community Health, Faculty of Public Health, Kasetsart University, Chalermphrakiat Sakonnakhon Province Campus, Sakonnakhon Province Thailand; 3 Buengkhonglong Hospital, Buengkan Province, Thailand

**Keywords:** Oral health knowledge, Oral health literacy, Oral health behavior, Pregnant women, Qualitative research

## Abstract

**Background:**

Pregnancy is a unique period of women’s lives, and oral health is an important public health indicator during this period. Pregnant women have increased vulnerability to oral health problems. The study aimed to describe oral health knowledge, literacy and behavior of pregnant women in a northeastern province of Thailand.

**Methods:**

A descriptive study was used. Twenty pregnant women who attended antenatal care clinics of eight public hospitals in the province were recruited by use of purposive sampling. They participated voluntarily in individual interview. The Health Belief Model was used as conception framework. All data were transcribed and subjected to content analysis.

**Results:**

Five categories emerged: Misbelief and lack of knowledge, Oral health problems and dental care seeking, Oral health information from different persons, Self-care management of oral health, and Fear of and anxiety towards dental treatment. The findings showed that low knowledge of need for treatment, little importance to oral health and low priority of dental needs affect the demand for dental care. Fear of and anxiety towards dental treatment were the results of negative past experiences of neglecting dental care. Some women perceived health benefits of practicing self-care of oral health during pregnancy.

**Conclusion:**

The findings help to better understand the oral health issues of pregnant women and provide baseline information for oral health promotion. Such promotion and culturally appropriate care should be integrated in maternal health education classes.

## Background

Oral health is an important public health indicator during pregnancy [1]. Pregnant women have increased vulnerability to oral health problems, e.g., gingivitis, gingival lesions, tooth mobility, tooth erosion, dental caries, and periodontitis [2–4]. Pregnancy gingivitis usually reaches its highest level during the eighth month of gestation and heals spontaneously after delivery [4]. The changes are associated with physiological increase in estrogenic hormone levels, poor oral hygiene practices, microbial changes in oral flora, daily diet alterations, frequent snacking, and vomiting [3, 5, 6]. Poor oral health during pregnancy has been associated with increased risk of pre-term birth, low birth weight, clinical manifestation of pre-eclampsia [3, 7] and other health problems such as diabetes and cardiovascular disease [8, 9].

The most common barriers of pregnant women in Maryland [10] and Tehran [11] for seeking dental care were high treatment costs, and lack of insurance and of perceived need for dental treatment. Additional barriers were fetal safety, beliefs about effects of pregnancy on dental health, and unwillingness to receive dental care [12, 13].

Different cultures have different meanings of what constitutes health and what causes illness [14]. In this study, the Health Belief Model (HBM) was used as conception framework. According to the HBM, a person’s health behaviour can be explained by the individual’s beliefs’ in a health action. The HBM includes five dimensions as a basis for behaviour: perceived severity of the condition, perceived susceptibility or vulnerability, perceived benefits, costs/barriers, and cues to action. Health-seeking behavior is generally influenced by the individuals’ perceived susceptibility to the illness, by the perceived severity of the illness, and by the evaluation of benefits and barriers to action [15]. Factors associated with the individual’s behavior include low education levels, low health-care knowledge, cultural customs, distrust in the healthcare system, and pre-existing false beliefs [16, 17].

Knowledge of oral health is limited, and many misconceptions are perceived by pregnant women. Many middle- and high-income countries have implemented strategies to improve pregnant women’s oral health, which include use of prenatal care providers [18]. In high- and middle-income countries such as Thailand, oral health is accepted as an important component of the general well-being of pregnant women. Therefore, the Thai Ministry of Public Health and the Thai National Health Security Office [19] have offered pregnant women oral health examinations and treatments free of charge.

Previous quantitative studies in Thailand showed that pregnant women had oral health problems, e.g. dental caries, plaque, gingivitis, and periodontitis [20–22]. Low education, age, inadequate oral health care, poor oral hygiene, and lack of knowledge were risks for oral health problems during pregnancy [20, 21, 23]. However, little knowledge about oral health problems of pregnant women has been gained by use of qualitative method. The results of such studies could be used as baseline information for an oral health education program aimed at improving the oral health care of pregnant women during their visits at antenatal care services. Therefore, the aim of this study was to describe the oral health knowledge, literacy and behavior of Thai pregnant women in a northeastern province of Thailand.

## Methods

### Design

A qualitative descriptive study with content analysis by using semi-structured interviews was conducted. The use of qualitative method provided in-depth and rich information that helped to understand the oral health knowledge, literacy and behavior of women during pregnancy [24]. The study was carried out in Bueng Kan, one of the upper northeastern provinces of Thailand. This province has a mixture of residential areas (i.e. rural and semi-urban), has eight government hospitals and borders the Mekong river and the Lao People’s Democratic Republic. Annually, approximately 1200 pregnant women visit the antenatal care (ANC) clinics of these hospitals [25]. Data were collected from May to September 2022.

### Participants

The participants were selected by use of purposive sampling from a list of pregnant women visiting the ANC clinics of all hospitals [20]. The inclusion criteria were pregnant women: (1) of age at least 18 years, (2) having 8–26 weeks of gestation based on data from their last menstrual period or ultrasound history in the maternal and child diary book (ANC Pink Book), (3) attending ANC clinics at the eight hospitals at the time of data collection, (4) being native Thais, and (5) being willing to participate. The exclusion criteria were pregnant women: (1) requiring emergency care or having illness that requires hospitalization, surgery, or treatment of severe infections, (2) having a history of mental diseases, and (3) having migrated from other countries.

### Ethical considerations

The study was approved by Kasetsart University Chalermphrakiat Sakonnakhon Province Campus Research Ethics Committee in Thailand (COA65/008), and was conducted in compliance with the ethical requirements outlined in the Declaration of Helsinki [26]. The participants, fully informed about the purpose of the study, assured of anonymity and confidentiality, and told that anyone could drop out at any time, had given informed consent verbally and in writing. Alphabetical codes were used to identify the transcribed data.

### Data collection

An interview guide was developed. The open-ended interview started with demographic questions followed by seven questions concerning oral health knowledge, literacy and behaviour of pregnant women: (1) How do you feel about your current pregnancy? (2) How do you describe your dental and oral status before and during pregnancy? (3) How do you evaluate your oral health knowledge? (4) What do you do about your oral health care before and during pregnancy? (5) How do you deal with dental and oral health problems? (6) What is your opinion about visiting the dentist for a dental checkup during pregnancy? (7) Is there anything else you would like to tell? The questions were tested with three women fulfilling the inclusion criteria but not participating in the study. The results showed that the interview guide was adequate.

### Procedure

The study started after approval of the directors of the hospitals. The pregnant women were referred by the nurses/midwives to dental clinics for screening of oral health status during their visits at the antenatal care clinics of the hospitals. After having received a list of pregnant women from the nurses/midwives, the researchers asked the women if they were willing to participate in an individual interview. The women received written and oral information before signing a written consent form. They were invited to a dental unit for oral examination and evaluation of oral parameters by a dentist who coded the parameters and recorded them in the participants’ ANC Pink Book. Thereafter, the women were interviewed in a room of the dental clinic of each hospital for 30–45 min. Sometimes they were asked to clarify. While they narrated their experiences, their facial expressions and gestures were observed and noted. The interviews were audio recorded and continued until no new information emerged. Twenty-three pregnant women with different demographic characteristics were contacted and invited to participate. As saturation had been reached after 20 interviews, however, the remaining three women were not interviewed. The recorded interviews were transcribed verbatim.

### Data analysis

The data from the interviews were analysed by use of qualitative content analysis [24]. This method is well suited for analyzing the multifaceted, important and sensitive oral health knowledge, literacy and behavior of pregnant women during pregnancy. The researchers carried out the data analysis separately, and then the findings were checked and discussed together. The transcripts were read repeatedly to obtain overall sense and make reflective notes. The analysis began by highlighting sentences of importance and dividing them into meaning units. The meaning units were condensed and labelled with short codes. Thereafter, the codes were compared to identify similarities and differences. Categories were developed based on the codes which included manifest content. Finally, the emerging categories were tested and revised through analyses of the interviews. The outcomes were discussed and modified to ensure reliability. Peer checking, validation of emerging codes and categories, and debriefing by an external researcher of the research methods and the subject were used to enhance credibility. Disagreements among the researchers were discussed until consensus was reached [27].

Trustworthiness [28] was addressed in several steps: credibility, confirmability, dependability, and transferability. Credibility was achieved by interviewing participants with various characteristics in terms of age, civil status, education and gestations, and the results were discussed. Confirmability was strengthened by using an external researcher who was expert in qualitative methods and by letting participants recheck their answers during the interviews. The use of tested interview guides, field notes and separate analyses increased the dependability. Transferability was assured by providing thorough descriptions of the contents and methods.

## Results

Twenty pregnant women were interviewed. Their age range was 18 to 43 years, and they lived in rural areas. Half the number of women were unemployed. Although the majority had oral health problems, they had not used dental service during the last six months. The demographic characteristics of the women are presented in Table 1.

**Table 1:** Demographic characteristics of pregnant women (*N* = 20)

Characteristics	Number (%)
Age: Median (range) = 26 (18–43) <20 years 20 years or more	2 (10) 18 (90)
Education Secondary School or lower High School or more	8 (40) 12 (60)
Occupation Unemployed Agriculturist Daily worker Self-employer	10 (50) 5 (25) 3 (15) 2 (10)
Residential area Rural Urban	20 (100) 0 (0)
Religion Buddhist Christian	20 (100) 0 (0)
Marital status Live together Not together	18 (90) 2 (10)
Type of family Nuclear family Extended family	15 (75) 5 (25)
Gravida status Primigravida Multigravida	11 (60) 9 (40)
Dental service history (the last 6 months) No Yes (Filling/Scaling/Extraction/Surgical removal of the wisdom tooth)	15 (75) 17 (85)
Dental caries status No Yes	15 (75) 5 (25)
Gingivitis status No Yes	1 (5) 19 (95)
Dental calculus status No Yes	1 (5) 19 (95)

Five categories of oral health knowledge, literacy and behavior of the pregnant women emerged: Misbelief and lack of knowledge, Oral health problems related to dental care seeking, oral health information from different persons, Self-care management of oral health, and Fear of and anxiety towards dental treatment.

### Misbelief and lack of knowledge

Most of the pregnant women mentioned several misbeliefs about suitable oral health behavior. They used garlic and salt to put into their teeth for reducing toothache. They believed that frequent brushing during pregnancy results in weakened teeth due to loss of calcium. Some women believed that dental treatment during pregnancy is unsafe and going to harm their unborn babies. Some women believed that they had tooth problems because of inheritance from their fathers. They were also told by their mothers that toothache came from the inside of their brains and this could result in early childbirth. Also, they believed that toothpaste with herbal remedies would reduce their tooth problems, e.g., toothache, gingival inflammation and oral malodor. They believed that this type of toothpaste when put into their teeth could help them reduce severe pain.

*I bought toothpaste from the market. The seller told me that it would make my teeth white. I put this toothpaste into my teeth when I had toothache and had swelling gum. I felt better after three days. …but now I could not find this toothpaste anymore. (Woman aged 20 years, 1st pregnancy, and 15 weeks of gestation)*

Some women used the wrong method for oral health, such as using needle or small pincers to take away caries between their teeth. Also, a few women used toothpick to remove food from the teeth and this caused broken teeth.

*… I usually use a needle to remove caries little by little between my teeth. It is sharp to use. I use it carefully to prevent bleeding. (Woman aged 33 years, 3rd pregnancy, and 21 weeks of gestation)*

The pregnant women had a lack of knowledge of oral hygiene. They did not know that they could have increased risk of oral disease during pregnancy. They were also unaware of the possibility of preventive dental visits before and during pregnancy and the advantage of fluoride in preventing dental decay. Therefore, they preferred to delay or restrict their dental treatment. Some experienced pregnant women described that the dentist and the dental assistant had told them that the unborn babies absorb calcium from the bones and teeth of their mothers due to hormone change.

*It is not necessary to visit the dental care service during pregnancy, particularly when I have toothache, because the dentist will not do anything when I have pain. (Woman aged 33 years, 3rd pregnancy and 10 weeks of gestation)*

### Oral health problems related to dental care seeking

The women described what they faced in their daily-life situation during pregnancy related to oral health care. This category consisted of two sub-categories: Oral health problems related to physiological changes, and Visiting dental care service.

### Oral health problems related to physiological changes

Physiological changes of the pregnant women, such as nausea and vomiting from morning sickness, were the reason that they used less time for teeth brushing than before pregnancy. A few women had more tooth problems during than before pregnancy. Some women did not have any tooth problems because they had been examined and treated by a dentist every time they visited the maternal health services at the ANC clinics.

*… The dentist found that I had dental caries in my front teeth, and I knew that I did not have this problem before pregnancy. She told me that I need to treat them…so I followed her recommendation… (Woman aged 18 years, 1st pregnancy and 15 weeks of gestation)*.

The women experienced various oral health problems during pregnancy, e.g., gingivitis, dental caries, malodor, and spots in the mouth. Most of the women did not observe any change in their mouths. Some women suffered from dental decay and loss of teeth. Other problems during pregnancy such as gum bleeding and toothache were also common among them.

*I had swelling of my gum and bleeding during 3–4 months of pregnancy. The dentist told me to pay attention to oral hygiene. (Woman aged 33 years, 2nd pregnancy and 10 weeks of gestation)*

### Visiting dental care service

Little importance was given to oral health, and low priority of needs for dental care was expressed. Many women used less dental services even though they had been informed that free dental care was available in their communities. The women usually decided to visit the dentists when they had tooth problems. Those who had experiences of oral health tended to appreciate dental visits both for themselves and for their children. Some women described that limitation of movements when sitting in the dental chair prevented them from seeking dental care.

*If I have not had toothache, I will not go to see the dentist… I feel toothache when I drink cold water so I have to visit the dental care service after visiting ANC … and the dentist told me that I have tooth decay during pregnancy and need to be treated. (Woman aged 22 years, 2nd pregnancy and 16 weeks of gestation)*

### Oral health information from different persons

Most of the pregnant women described that family and friends advised them to solve tooth problems such as toothache by taking a pain-reducing drug before visiting the dentist. Some women mentioned that they followed the recommendation of the dentist or the doctor not to buy or take medicine without consulting a dentist or the prescription of a doctor. They received support from family, partners and friends who brought them to dental clinics of the hospitals when they needed help. Also, they received information from the dentists that improper oral health behavior among the pregnant women could cause plaque deposit and oral malodor, and tooth infection could go to their fetus by saliva and food. Moreover, experiences from their previous pregnancy had effect on their oral health behavior.

*My sister-in-law told me that I can take paracetamol to reduce toothache before visiting the dentist. (Woman aged 20 years, 1st pregnancy and 15 weeks of gestation)*

*I did not have so good experience of oral health care during my first pregnancy. This time my family, for example my husband, my parents and my parents-in-law, supported me and brought me to visit the dental care service and consult the dentist about the tooth root. (Woman aged 32 years, 2nd pregnancy and 20 weeks of gestation)*

Most of the women searched information about oral health care of pregnant women from the Internet, e.g. google, face book, and App. They were informed that pregnant women could repair their teeth after four months of pregnancy because the fetus was formed during a period of one to three months. The advertisement on TV and books such as the ANC pink book were used to select toothpaste.

*I look at my mobile phone to search online information from YouTube what I could do when I have tooth problems. … (Woman aged 20 years, 1st pregnancy and 15 weeks of gestation)*.

### Self-care management of oral health

Self-care management of oral health was a good habit. Regular tooth brushing in the morning and before bedtime was expressed as their self-care to prevent dental caries and oral malodor. All women followed the advice from dentists to treat dental decay and brush the teeth twice daily. Some women also brushed their tongue. A few women also used dental floss or mouthwash. The women consulted dentists at the dental care clinics about oral health problems to get dental treatment, e.g., tooth filling, caries-frees, and tooth decay.

*I brush my teeth two times per day. Also, I use dental flossing and mouthwash to clean once per day before going to bed. (Woman aged 27 years, 1st pregnancy and 23 weeks of gestation)*

*I received dental treatment when I felt that my teeth were obstructed by caries because the water could not flow out. I thought my teeth had accumulated caries. It looked like a wall between the teeth. (Woman aged 33 years, 2nd pregnancy and 10 weeks of gestation)*

### Fear of and anxiety towards dental treatment

Fear of and anxiety towards dental treatment were the result of negative past experiences of the pregnant women. Many women felt fear and anxiety to go to a dentist since their childhoods. They were afraid that repairing their teeth could affect their fetus. Some of them mentioned that they felt fear and were excited when they had to receive dental treatment because they had not visited the dentist for a long time.

*I have felt fear to visit the dentist from childhood until now. I felt pain while the dentist filled a tooth. I was worried each time when I had to visit a dentist. (Woman aged 18 years, 1st pregnancy and 15 weeks of gestation)*

## Discussion

This study highlights misbeliefs about oral health that were common among the pregnant women. This agrees with findings of other studies [8, 29, 30] showing that lack of information about oral health during pregnancy results in misbeliefs and myths about safety of dental treatment. Moreover, it shows that the women had lack of oral health knowledge. This is in line with other studies [12, 20, 21, 23, 31]. Poor oral hygiene could be prevented if the pregnant women acquired adequate knowledge and adopted appropriate daily hygiene practices [11, 32, 33]. According to HBM [15], the women did not perceive the severity and the complications of oral health problems. It seems that misbelief and lack of knowledge were barriers for oral health care. Dental prenatal care could also encompass women’s health and aim to clarify myths and beliefs regarding the safety of dental treatment during pregnancy [11, 12]. Therefore, it is important to integrate oral health topics including culturally appropriate care in information given to prenatal classes. Furthermore, current guidelines on oral health counseling, screening and referral to a dental clinic during pregnancy should be in focus of prenatal visits.

The participating women’s symptoms of morning sickness from pregnancy resulted in shorter teeth brushing. The physiological changes during pregnancy may aggravate dental and periodontal diseases through hormonal changes and alterations in eating habits [31, 34]. Increased pH of the oral cavity due to vomiting, frequent meals rich in carbohydrates, and poor oral hygiene contributes to the development of caries during pregnancy. If left untreated, it may lead to local and systemic complications [35].

Inconsistent use of dental care contributed to low awareness of the importance of oral health and appropriate oral hygiene of the participating women. Even though free dental care was available in their communities [19], they usually did not visit a dentist until they had tooth problems. This behavior is supported by previous studies [31, 36] showing that pregnant women were unaware of the importance of dental health care. The policy of the Thai Ministry of Public Health is that around 30% of pregnant women should receive oral health examinations, and oral health promotion and prevention services should be offered to at least 70% of pregnant women [19]. Additionally, according to the dental services report for the strategic planning of oral health standards in Bueng Kan province, the prevalence of dental caries in pregnant women was 74% while only 22% of pregnant women received oral health checks and care [37]. Oral health examination was carried out as a part of routine check-ups as indicated in the guideline for pregnant women from the Ministry of Public Health, Thailand [25]. Pregnant women should be motivated to pay attention to regular oral health examinations when participating in prenatal classes of the maternal health education program.

Various oral health problems during pregnancy were experienced. Little attention given to oral health and low priority given to dental needs affected the participating women’s attendance to dental care. Education level, income, residential area, culture, and belief in advertising media might have been barriers for the women’s interest in oral health care behavior. Dental care seeking behavior is influenced by the women’s perceived susceptibility to dental diseases, by the perceived severity of the diseases, and by the evaluation of benefits and barriers to actions of self-care [15]. Basic oral hygiene education may improve oral health and contribute to improving the quality of life among pregnant women [38]. Regular dental service utilization can assist in early detection of oral problems and improve oral health status [2, 11, 39]. Therefore, pregnant women should be given knowledge about oral health and dental care in order to change their attitudes towards dental service utilization.

Family and friends advised the participating women to buy a drug reducing their tooth pain before visiting the dentist. It is in accord with other studies [8, 11] that family and friends may contribute to barriers by giving misleading advice regarding safety of treatment based on their own experiences. Some women were supported by family, partners and friends. Women who received financial and psychological support from their partners and family sought dental care more often than those who did not [40]. Pregnancy is thought to be an important period for imparting oral health information and supporting women to adopt positive oral health behaviors [41].

Self-care management of oral health by the participating women focused on their daily oral health hygiene. Appropriate knowledge, attitude and behavior of pregnant women could prevent oral health problems and complications during pregnancy [42, 43]. In accord with Becker [15], the women had increased their chances of successful self-care management by believing in the health benefits of oral health control. Oral health for pregnant women has been integrated into public health services and primary health care [44]. Worldwide, women still show low dental service utilization. Therefore, it is important to change their attitude towards dental attendance during pregnancy [45]. Pregnant women should receive comprehensive oral health education, evaluation and referral to dentists in case of treatment needs.

Fear of and anxiety towards dental treatment were results of negative past experiences that inhibited the participating women from visiting the dentist during pregnancy. Fear of pain, dental procedures and the environment of dental clinics were also cited. This is in line with other studies [2, 46, 47] which found an association between dental fear and dental treatment seeking. Perceived stress and anxiety and low self-efficacy might lead to neglect of oral hygiene and deterioration of periodontal health during pregnancy [48]. Lack of insurance coverage for dentistry expenses and of time to visit the dentist were major barriers of the women [49]. Dental treatment during pregnancy might have a negative effect on pregnancy outcome and be an important factor limiting the utilization of dental care. To minimize fear and anxiety regarding dental care for pregnant women, prenatal dentistry should focus on health literacy, creation of space for dialogue and exchange of knowledge.

This study was aimed at describing oral health knowledge, literacy and behavior among pregnant women. We used qualitative method to obtain rich data for understanding of these issues in a north-eastern Thai context. The study was conducted in Bueng Kan, a province of upper northeastern Thailand, which limits the transferability to other settings. Therefore, the findings may not represent other provinces in the region or other contexts. Pregnant women might have different experiences in other cultural and socioeconomic environments. In addition, some women may have felt it difficult to describe their experiences. However, the in-depth interviews gave increased understanding of the pregnant women’s experiences through their own words. Additionally, the results are valuable as a basis for further investigations aimed at developing an intervention program of prenatal oral health education for pregnant women visiting maternal care services at the hospitals.

## Conclusion

The findings highlight that misbelief and low knowledge of need for treatment, little attention to oral health and low priority of dental needs affect the demand for dental care. Fear and anxiety towards dental treatment were results of the pregnant women’s negative past experiences of dental visits. Support from their partners, family and friends were expressed. They realized health benefit by practicing self-care of oral health during pregnancy. The findings could help to better understand oral health issues of pregnant women and provide baseline information for oral health promotion. Oral health promotion should be included in maternal health education classes by integrating oral health topics including culturally appropriate care. Pregnant women should receive comprehensive oral health education and referral to dentists in case of treatment needs.

## Data Availability

The datasets used and/or analysed during the current study are available from the corresponding author on reasonable request.

## Abbreviations

HBM: Health Belief Model
ANC: Antenatal Care
