# Correction: Oral health knowledge, literacy and behavior of pregnant women: a qualitative study in a northeastern province of Thailand

**DOI:** 10.1186/s12903-024-04930-2

**Published:** 2024-09-23

**Authors:** Nitikorn Phoosuwan, Pimchanok Bunnatee, Pranee C. Lundberg

**Affiliations:** 1 Department of Public Health and Caring Sciences, Faculty of Medicine, Uppsala University, Box 564, Uppsala, SE-751 22 Sweden; 2 Department of Community Health, Faculty of Public Health, Kasetsart University, Chalermphrakiat Sakonnakhon Province Campus, Sakonnakhon Province, Thailand; 3 Buengkhonglong Hospital, Buengkan Province, Thailand

**Correction to: BMC Oral Health (2024) 24:653**

10.1186/s12903-024-04414-3

In this article [1], due to misalignment of entries, Table 1 is incorrect throughout. The corrected Table 1 is given below.

**Table 1:** Demographic characteristics of pregnant women (*N* = 20)

Characteristics	Number (%)
Age: Median (range) = 26 (18–43)	
<20 years	2 (10)
20 years or more	18 (90)
Education	
Secondary School or lower	8 (40)
High School or more	12 (60)
Occupation	
Unemployed	10 (50)
Agriculturist	5 (25)
Daily worker	3 (15)
Self-employer	2 (10)
Residential area	
Rural	20 (100)
Urban	0 (0)
Religion	
Buddhist	20 (100)
Christian	0 (0)
Marital status	
Live together	18 (90)
Not together	2 (10)
Type of family	
Nuclear family	15 (75)
Extended family	5 (25)
Gravida status	
Primigravida	11 (60)
Multigravida	9 (40)
Dental service history (the last 6 months)	
No	15 (75)
Yes (Filling/Scaling/Extraction/Surgical removal of the wisdom tooth)	17 (85)
Dental caries status	
No	15 (75)
Yes	5 (25)
Gingivitis status	
No	1 (5)
Yes	19 (95)
Dental calculus status	
No	1 (5)
Yes	19 (95)
